# Longitudinal national surveys reflect steady challenges for AIDS drug assistance programs, 2017–2024

**DOI:** 10.3389/fpubh.2026.1913359

**Published:** 2026-08-26

**Authors:** Erin Q. Rogers, Amy Killelea, Tim Horn, Reanna Panagides, Jessica Keim-Malpass, Feng Liu, Amber Steen, Kathleen A. McManus

**Affiliations:** 1 Division of Infectious Diseases and International Health, University of Virginia School of Medicine, Charlottesville, VA, United States; 2 McCourt School of Public Policy, Georgetown University, Washington, DC, United States; 3 NASTAD, Washington, DC, United States; 4 University of Virginia School of Nursing, Charlottesville, VA, United States

**Keywords:** Affordable Care Act, AIDS Drug Assistance Program, HIV, HIV medications, insurance assistance, Medicaid, public health practice, Ryan White HIV/AIDS Program

## Abstract

As part of the Ryan White HIV/AIDS Program, AIDS Drug Assistance Programs (ADAPs) provide HIV medications and insurance assistance to low-income people with HIV across all U. S. states, the District of Columbia, and territories. The share of clients receiving insurance assistance through ADAPs has grown considerably, particularly following the Affordable Care Act (ACA), requiring ADAPs to coordinate with an increasingly complex array of public and private payers. This is the first study to examine longitudinal trends in the challenges ADAPs report. We conducted a descriptive analysis of the challenges reported by ADAPs between 2017 and 2024 in the NASTAD National ADAP Monitoring Project Annual Reports. ADAP leaders rated seven insurance-related domains (2017–2024) and 10 general program domains (2021–2024) as Not, Somewhat, and Very Challenging. We categorized responses as “Not Challenging” or “Challenging” (Somewhat/Very). Between 46 and 51 ADAPs responded annually. Although the overall number of domains rated as challenging decreased over time, six were rated as challenging by >50% of ADAPs in every year assessed: mail order pharmacy limitations, client churn, high copay/coinsurance, maintenance of eligibility, IT and data-sharing issues, and staff turnover. Some challenges increased, including pharmacy network issues (15 to 27%; +12 percentage points, +78% relative), while others declined, including remote work (37 to 10%; −27 percentage points, −72% relative). Despite a modest overall decrease in reported challenges, insurance coordination and core-operational challenges persisted across the years assessed. Continued monitoring may be particularly important as anticipated Medicaid and Marketplace changes take effect.

## 1 Introduction

As part of the Ryan White HIV/AIDS Program (RWHAP), AIDS Drug Assistance Programs (ADAPs) provide access to HIV medications for low-income people with HIV (PWH) in every U. S. state, the District of Columbia (DC), and the territories. ADAPs are administered by state and territorial health departments and receive federal, and in some jurisdictions, state funding. ADAPs assist clients in two primary ways: they purchase and distribute medications for clients without another source of coverage, and they provide insurance assistance to pay premiums and medication copayments for insured clients.

Over time, the number of insured clients ADAPs serve has increased, particularly as more coverage options became available through the ACA (1). This shift has required ADAPs to navigate more complex policies and administrative systems to coordinate benefits, assess the cost-effectiveness of insurance plans, and manage payments with a range of public and private payers. Successful navigation enables ADAPs to serve an increasing number of clients each year despite relatively flat RWHAP funding (2). At the same time, increased insurance purchasing and coordination have heightened ADAPs’ exposure to changes in Medicaid eligibility and benefits, Marketplace policies, insurer participation, premiums, cost-sharing requirements, formularies, and other plan-design features. These changes may create recurring or evolving administrative and financial challenges that affect program operations and clients’ continuity of coverage and medication access.

However, longitudinal trends in the challenges reported by ADAPs as insurance coordination has become more complex have not been evaluated. Specifically, it is not known whether the challenges have persisted, intensified, or eased over time. Understanding these historical trends is relevant to future program planning, particularly given anticipated changes to Medicaid and the health insurance Marketplace.

To describe the challenges ADAPs have faced and how they have changed over time, we examined survey responses from ADAP leaders compiled in the NASTAD National ADAP Monitoring Project Annual Reports from 2017 to 2024. These findings may help state ADAPs, state health departments, and the Health Resources and Services Administration (HRSA) HIV/AIDS Bureau identify persistent and emerging challenges, inform funding and technical-assistance priorities, or determine where continued monitoring may be beneficial.

## 2 Materials and methods

**Figure 1:**
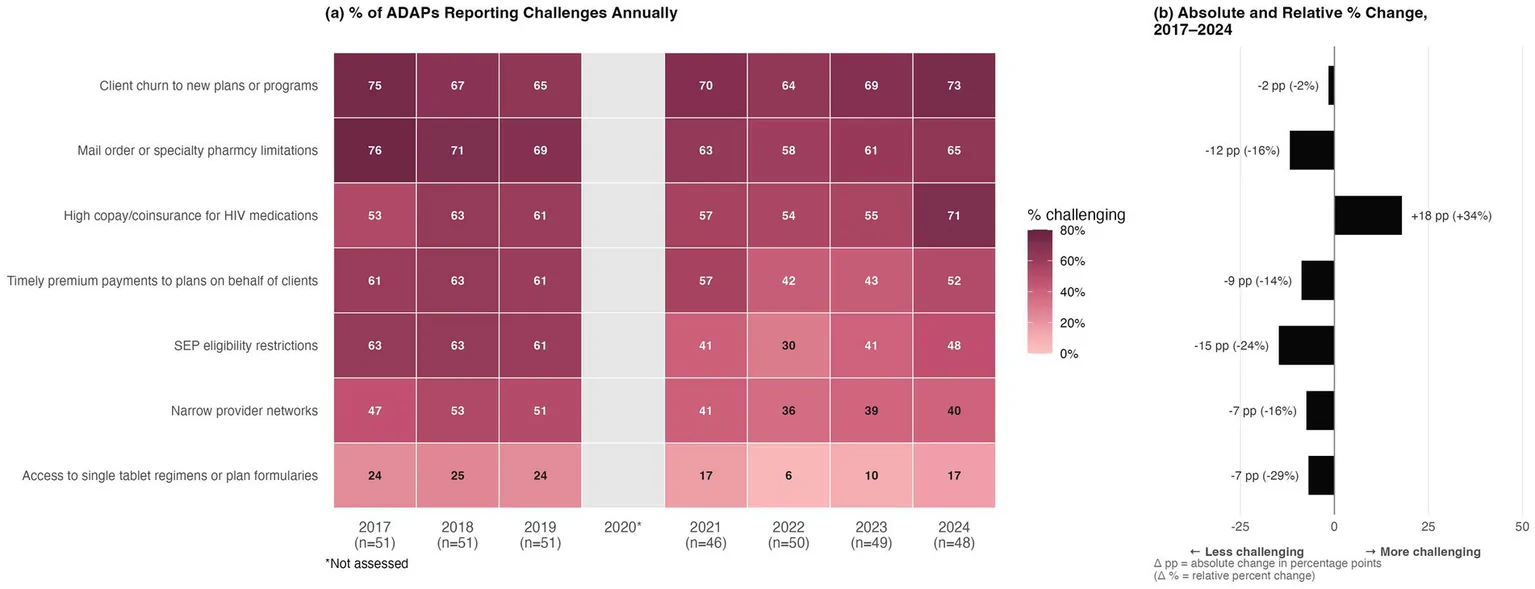
Prevalence of ADAP-funded insurance program challenges reported by U. S. AIDS drug assistance programs (ADAPs), 2017-2024. SEP, Special enrollment period. All 50 states and the district of Columbia participated in the survey in 2017, 2018, and 2019. In 2021, Alabama, Maryland, Mississippi, Virginia, and West Virginia did not participate. In 2022, West Virginia did not participate. In 2023, Alabama and West Virginia did not participate. In 2024, West Virginia and South Dakota, and Mississippi did not participate. Survey data was not collected in 2020.

Data were obtained from jurisdiction-level survey responses collected through the 2019–2026 NASTAD National ADAP Monitoring Project Annual Reports, covering 2017–2024 (1, 3–6). Challenges were not collected for 2020. The National RWHAP Part B ADAP Monitoring Project is an annual survey administered by NASTAD to all RWHAP Part B/ADAP jurisdictions. Jurisdiction program staff voluntarily report information on program operations and challenges. The unit of analysis was the individual responding jurisdiction. The eligible jurisdictions included the 50 states and the District of Columbia; U. S. territories were not included in these analyses (Figure 1).

**Figure 2:**
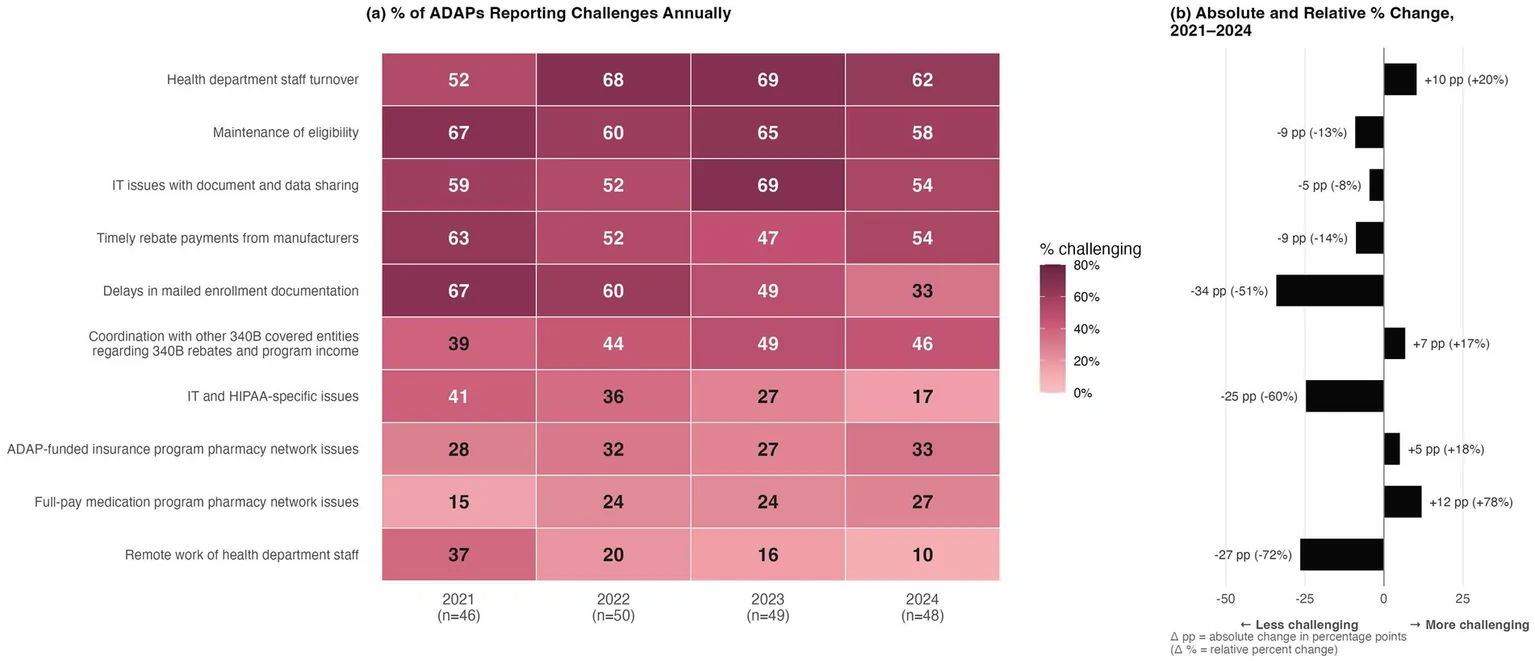
Prevalence of ADAP-funded insurance program challenges reported by U. S. AIDS drug assistance programs (ADAPs), 2021-2024. HIPAA, Health insurance portability and accountability act; ADAP, AIDS drug assistance program; IT, Information technology. In 2021, Alabama, Maryland, Mississippi, Virginia, and West Virginia did not participate. In 2022, West Virginia did not participate. In 2023, Alabama and West Virginia did not participate. In 2024, West Virginia and South Dakota, and Mississippi did not participate.

ADAP-funded insurance program challenges were assessed throughout the study period from 2017 to 2024. General ADAP program challenges were added in 2021 to assess the effects of the COVID-19 pandemic on programmatic processes and continued to be assessed through 2024. Each challenge was measured using a single survey item, referred to here as a domain because the survey items assessed broad thematic areas of program operations. Seven ADAP-funded insurance program domains were assessed from 2017 to 2024: single tablet regimen access, high copay or coinsurance, mail order or specialty pharmacy limitations, timely premium payments, client churn, narrow provider networks, and special enrollment period eligibility restrictions. Ten general ADAP program domains were assessed from 2021 to 2024: maintenance of eligibility, delays in mailed documentation, IT and data-sharing issues, staff turnover, remote work, HIPAA-specific issues, timely manufacturer rebates, direct provision medication program pharmacy network issues, ADAP-funded insurance program pharmacy network issues, and coordination with other 340B covered entities (Figure 2).

ADAP jurisdiction staff rated each domain as Not Challenging, Somewhat Challenging, or Very Challenging, or select Not Applicable. Because the objective was to identify jurisdictions reporting any degree of challenge, we combined Somewhat Challenging and Very Challenging into a single “Challenging” category. This dichotomization does not distinguish severity levels represented by the original response categories. We grouped Not Challenging and Not Applicable responses into a “Not Challenging” category, based on the assumption that a Not Applicable response indicated that the domain was not relevant to the jurisdiction and therefore was not experienced as a challenge.

Because responding jurisdictions varied somewhat across years, annual percentages were calculated among jurisdictions responding in each year. Observed changes may therefore reflect differences in the composition of respondents as well as changes in program experiences. For each domain, we calculated the percentage of responding jurisdictions rating it challenging in each year. We summarized changes between the first and last year of assessment: 2017 to 2024 for ADAP-funded insurance program challenges and 2021 to 2024 for general program challenges using both absolute percentage-point change and relative percent change. Absolute percentage-point change was calculated as the final-year percentage minus the initial-year percentage. Relative percent change was calculated as the absolute percentage-point change divided by the initial-year percentage and multiplied by 100.

Analyses were descriptive; no inferential testing, trend modeling, or uncertainty estimation was performed. Jurisdiction-level characteristics were not examined.

## 3 Results

Data were submitted by 46–51 ADAPs (states and the District of Columbia) annually. For 2017 to 2019, the survey response rate was 100, 90% for 2021, 98% for 2022, 96% for 2023, and 94% for 2024. Among the seven insurance program domains assessed from 2017 to 2024, the mean number of domains rated as challenging by ADAPs decreased from 3.98 (SD 1.95) in 2017 to 3.65 (SD 1.80) in 2024. Among the 10 general program domains assessed from 2021 to 2024, the mean number of domains rated as challenging by ADAPs decreased from 4.70 (SD 2.58) in 2021 to 3.96 (SD 2.18) in 2024. Despite this small improvement, six domains were rated as challenging by over 50% of ADAPs each year that the domain was assessed – three insurance program domains (mail order/specialty pharmacy limitations, client churn, and high copay/coinsurance) and three general program domains (maintenance of eligibility, IT/data-sharing issues, and staff turnover).

States reported increasing challenges over the study period across a range of issues, some related to insurance purchasing and some implicating ADAP operations more broadly. Challenges regarding issues related to the direct provision medication program pharmacy networks, coordination with other 340B covered entities, high copay/coinsurance for HIV medications, and health department staff turnover all increased over the study period, with increases ranging from 17 to 78%. States reporting staff turnover increased from 52% in 2021 to 62% in 2024 (+20% relative) even after states no longer experienced the same pandemic-related stressors of 2021. And finally, problems with client churn to new plans or programs – where clients may enroll and disenroll in ADAP over the course of a year because they are eligible for other programs – remained a consistent challenge over the course of the study period.

In contrast, other challenges showed signs of improvement over the study period. Navigating special enrollment period eligibility restrictions (SEPs; which insurance plans use to allow individuals to enroll outside of an open enrollment period if they experienced a qualifying life event), navigating insurance pharmacy mail order and specialty pharmacy limitations, and ensuring ADAP ability to make timely premium payments on behalf of insured clients all decreased over time in absolute and relative terms (SEP restrictions: 63 to 48%, relative −24%; mail order/specialty limitations: 76 to 65%, relative −16%; timely premium payments: 61 to 52%, relative −14%). Importantly, the domain that had the lowest percent of ADAPs rate it as challenging in all years was access to single tablet regimens (STRs) (2017: 24%; 2024: 17%), with a 29% relative decrease in states reporting this as a challenge over time.

Two programmatic challenges surveyed from 2021 to 2024 had the largest absolute percent change during the study period. The number of ADAPs reporting challenges related to remote work for health department staff decreased from 37% in 2021 to 10% in 2024 (−72% relative) while those reporting challenges related to pharmacy networks for the direct-provision program increased from 15% in 2021 to 27% in 2024 (+78% relative). Over time, fewer states reported challenges with IT and HIPAA-specific issues, delays in mailed enrollment documentation, timely rebate payments from manufacturers, maintenance of ADAP eligibility, and ADAP-funded insurance program pharmacy network issues (ranges from −8% to −72% decreases). While some reported challenges decreased over this study over this study period, others persisted or increased, with challenges remaining across both ADAP-funded insurance mechanisms and broader programmatic processes.

## 4 Discussion

Our findings suggest that while some ADAP challenges have eased over time, several challenges were reported as challenging by more than half of responding jurisdictions in every year of the study period. These findings are descriptive and reflect ADAP-reported challenges rather than measured program outcomes. Three out of the six most frequently reported challenges (mail order pharmacy requirements, client churn, and high medication cost sharing) are associated with ADAP-funded insurance programs, while the other three reflect broader program operations, underscoring that challenges are agnostic to program types. This finding likely reflects that coordinating with health insurance is complex and may raise more challenges than traditional direct provision medication programs (7, 8). The nature of these reported challenges also suggests that insurance policy changes might go a long way in alleviating ADAP program complexity.

Mail order pharmacy requirements, for instance, are common among insurers and are often a cost-saving measure for employers and plans. However, many PWH may prefer to use a brick-and-mortar pharmacy for privacy reasons (9). ADAPs may also prefer client use of brick-and-mortar pharmacies that are more likely to be in an ADAP pharmacy network than mail order pharmacies. Use of an ADAP network pharmacy can ease client medication access and help ADAPs to facilitate payment of the client’s cost sharing obligation. Although the ACA allows individual market and small group plans to opt out of mail order requirements, mail order and specialty pharmacy limitations remained among the most frequently reported challenges throughout the study period (76% in 2017 to 65% in 2024). This sustained burden suggests that ACA policy protections alone have not eliminated access barriers, highlighting the need for future research to evaluate specific administrative hurdles and awareness gaps.

Client churn is also an ongoing challenge that may become more pronounced as anticipated federal changes to Medicaid are implemented (10, 11). In July 2025, Congress passed a law that makes a number of changes to Medicaid, including implementing work requirements and removing eligibility for certain immigrant groups (12). This law is expected to cause 7.5 million Medicaid enrollees to lose coverage over the next 10 years (13). This is coupled with anticipated coverage loss in the individual market because of federal changes to marketplace subsidy amounts and coverage rules (14). These anticipated coverage changes postdate our 2017–2024 data and thus were not evaluated here; they are noted as context for why continued monitoring of client churn will be important. If they materialize, ADAPs may need to absorb newly uninsured clients into full-pay medication programs and help clients find other coverage.

And finally, high medication cost sharing in private insurance plan designs is likely to continue, especially as prices for ARVs continue to rise (15). During the study period, ADAPs were less concerned about inclusion of STRs on plan formularies, but even when plans cover these medications, the cost sharing charged is a challenge. Employers and private insurance plans are increasingly turning to deductibles and coinsurance as a way to address rising prescription drug costs, which foists more cost sharing onto consumers (16). Policy proposals that rein in the price of medications (17) or that require standardized plan designs that do not utilize deductibles and coinsurance could help alleviate this challenge for ADAP insurance assistance programs (18). This work may highlight priority topics for technical assistance or HRSA evaluation of policy and resources. Because our data captured ADAP-reported challenges rather than insurance benefit design of medication prices directly, they speak to ADAP perceptions of cost-sharing challenges rather than to changes in benefit design or drug prices themselves.

These implications can be grouped into broad categories: payer coordination, eligibility administration, workforce capacity, and data infrastructure. ADAPs are navigating the increasing complexity of insurance coordination against a backdrop of broader programmatic challenges, including persistent staff turnover (workforce capacity) that may make it difficult to ensure staff have appropriate insurance-related training, experience, and skills. Increasing challenges with IT/data systems may also exacerbate insurance challenges, as coordination with large private insurance companies can be time-consuming without the appropriate data platforms.

Consistent with the persistent cost-related challenges reported here, since the time of the study, ADAPs began considering cost containment measures (19) and subsequently implemented some of these measures, such as reduced formularies, tightened financial eligibility criteria, and pharmacy cost-management strategies (20). Given the evolving policy and implementation environment in which ADAPs operate, ongoing monitoring of emerging challenges will be critical to ensure that issues are addressed at the appropriate level—whether through federal or state policy action, targeted funding, regulatory adjustments, or technical assistance.

This study has several strengths. To our knowledge, it is the first to characterize longitudinal trends in ADAP-reported challenges, drawing on a standardized national survey instrument administered annually to nearly all state ADAPs (response rates 90–100%) over an eight-year period, which supports comparison across time and captures challenges spanning both insurance-assistance mechanisms and broader programmatic operations, though comparability is tempered by year-to-year differences in participating jurisdictions and respondents. This study has several limitations that should be balanced with these strengths when interpreting the findings. First, the data are based on the subjective assessments of state ADAP staff using a 3-point Likert scale, which may introduce response bias or varying interpretations of what constitutes a “challenge.” Because different staff members may have completed the survey across the eight-year study period, longitudinal consistency in reporting could be affected by individual perspectives and varying levels of institutional memory. The survey is completed by ADAP program staff, and the respondent’s role within a program may influence how challenges are perceived and rated; respondent role was not captured in these data. Additionally, while response rates were generally high, they were not 100% in all years, and several states, including West Virginia, South Dakota, and Mississippi, did not participate in certain cycles. This may introduce non-response bias, as programs facing the most significant administrative or staffing challenges may have had the least capacity to complete the survey. In addition, the analysis did not include format trend testing or uncertainty estimation and observed changes over time may reflect differences in respondents or participating jurisdictions as well as true changes in program experience. The survey also did not ask whether or how jurisdictions addressed reported challenges; ongoing qualitative work is characterizing the operational practices associated with program success (21). Finally, the absence of data from 2020 due to the COVID-19 pandemic created a gap in the longitudinal trend during a period of significant operational challenges.

As ADAPs confront an increasingly complex health care ecosystem, they have adapted and evolved over time. As more clients have insurance coverage, for example, ADAPs have adopted policies and operations to allow programs to provide insurance assistance to low-income clients (22). State and federal policies that ensure that ADAPs have the staff, data/IT infrastructure, and funding to adapt to a far more complex environment may be necessary to ensure ADAPs can continue and grow these programs. At the same time, policies that make public and private insurance coverage simpler, more affordable, and more accessible will likely also have a significant impact on ADAPs’ ability to provide continued insurance support. Continued monitoring may be warranted as coverage options and out-of-pocket and premium cost policies evolve, since such changes could add to the challenges ADAPs already report.

## Data Availability

Publicly available datasets were analyzed in this study. This data can be found at: https://nastad.org/adap-monitoring-project.

## References

[1] NASTAD. *National Ryan White HIV/AIDS Program Part B ADAP Monitoring Project Annual Report 2026*. NASTAD (2026).

[2] Kaiser Family Foundation. *The Ryan White HIV/AIDS Program: The Basics* (2020). Available online at: https://www.kff.org/hivaids/fact-sheet/the-ryan-white-hivaids-program-the-basics/ (Accessed Aug 19, 2025).

[3] National Alliance of State and Territorial AIDS Directors. *National Ryan White HIV/AIDS Program Part B and ADAP Monitoring Project: 2019 Annual Report* (2019). Available online at: https://publications.partbadap-2019.nastad.org/ (Accessed Aug 27, 2019).

[4] National Alliance of State and Territorial AIDS Directors. *National RWHAP Part B ADAP Monitoring Project Annual Report, 2023*. (2023).

[5] National Alliance of State and Territorial AIDS Directors. *2024 National RWHAP Part B ADAP Monitoring Project Annual Report* (2024). Available online at: https://nastad.org/2024-rwhap-part-b-adap-monitoring-report. (Accessed Mar 30, 2025).

[6] National Alliance of State and Territorial AIDS Directors. *National ADAP Monitoring Project: 2021–2022 Annual Report* (2023). Available online at: https://nastad.org/partb-adap-2021-2022-report. (Accessed Feb 22, 2018).

[7] McManus KA Schurman E An Z Van Hook R Keim-Malpass J Flickinger TE. Patient perspective of people with HIV who gained Medicaid through Medicaid expansion: a cross-sectional qualitative study. AIDS Res Hum Retrovir. (2021) 38:580–91. doi: 10.1089/AID.2021.0129, 34538069 PMC9297321

[8] McManus KA Killelea A Honeycutt E An Z Keim-Malpass J. Assisters succeed in insurance navigation for people living with HIV and people at increased risk of HIV in a complex coverage landscape. AIDS Res Hum Retrovir. (2020) 36:842–51. doi: 10.1089/AID.2020.0013, 32631076 PMC7548024

[9] McManus KA Debolt C Elwood S Saint-Surin T Winstead-Derlega C Brennan RO . Facilitators and barriers: clients’ perspective on the Virginia AIDS drug assistance program’s affordable care act implementation. AIDS Res Hum Retrovir. (2019) 35:734–45. doi: 10.1089/AID.2018.0254, 31146536 PMC6688112

[10] McManus KA Strumpf AM Killelea A Horn T Steen A An Z . Ryan white HIV/AIDS part B and AIDS drug assistance programs during COVID-19: safety net public health programs’ challenges and innovations. Front Public Health. (2023) 11:1172009. doi: 10.3389/fpubh.2023.1172009, 37583891 PMC10425265

[11] McManus K Rogers EQ Horn T Killelea A Keim Malpass J Steen A . *1052 - Longitudinal Enrollment and Churn in the AIDS Drug Assistance Program in 3 States, 2014–2021 CROI Conference* (2026). Available online at: https://www.croiconference.org/abstract/1043-2026/ (Accessed May 31, 2026).

[12] Congress. *H.R.1 - 119th Congress (2025–2026): One Big Beautiful Bill Act [Legislation]*. (2025). Available online at: https://www.congress.gov/bill/119th-congress/house-bill/1 (Accessed Nov 3, 2025).

[13] Burns A Ortaliza J Lo J Rae M Cox C. *How Will the 2025 Reconciliation Law Affect the Uninsured Rate in each State?* KFF (2025). Available online at: https://www.kff.org/uninsured/how-will-the-2025-reconciliation-law-affect-the-uninsured-rate-in-each-state/ (Accessed Nov 3, 2025).

[14] Center on Budget and Policy Priorities. *Center on Budget and Policy Priorities [Policy Brief]*. (2025). Available online at: https://www.cbpp.org/research/health/by-the-numbers-harmful-republican-megabill-will-take-health-coverage-away-from (Accessed Oct 29, 2025).

[15] McCann NC Horn TH Hyle EP Walensky RP. HIV antiretroviral therapy costs in the United States, 2012-2018. JAMA Intern Med. (2020) 180:601–3. doi: 10.1001/jamainternmed.2019.7108 32011622, 32011622 PMC7042880

[16] Vu K Cotter L Rae M. *Peterson-KFF Health System Tracker [Issue Brief/Chart Collection]* (2025). How much do people with employer plans spend out-of-pocket on cost-sharing? Available online at: https://www.healthsystemtracker.org/chart-collection/how-much-do-people-with-employer-plans-spend-out-of-pocket-on-cost-sharing/ (Accessed Oct 29, 2025).

[17] United States, President. *The White House [Executive order/Government Document]*. (2025). Available online at: https://www.whitehouse.gov/presidential-actions/2025/05/delivering-most-favored-nation-prescription-drug-pricing-to-american-patients/ (Accessed Oct 29, 2025).

[18] Center on Budget and Policy Priorities. *Center on Budget and Policy Priorities [Policy Report/Issue Brief]*. (2024). Available online at: https://www.cbpp.org/research/health/building-on-the-affordable-care-act-strategies-to-address-marketplace-enrollees (Accessed Oct 29, 2025).

[19] NASTAD. *AIDS Drug Assistance Program (ADAP) Cost Containment Considerations* (2025). Available online at: https://nastad.org/resources/aids-drug-assistance-program-adap-cost-containment-considerations (Accessed Feb 11, 2026).

[20] NASTAD. *ADAP Watch: February 2026 National Alliance of State and Territorial AIDS Directors (NASTAD)* (2026).

[21] McManus KA Rogers EQ Steen A Killelea A Horn T Panagides R . Characterizing AIDS drug assistance program practices and policies for sustained viral suppression using the consolidated framework for implementation research: protocol for a qualitative study. JMIR Res Protoc. (2026) 15:e90008. doi: 10.2196/90008, 41858071 PMC13133592

[22] McManus KA Rodney RC Rhodes A Bailey S Dillingham R. Affordable care act qualified health plan Enrollment for AIDS drug assistance program clients: Virginia’s experience and best practices. AIDS Res Hum Retrovir. (2016) 32:885–91. doi: 10.1089/AID.2016.0033, 27346694 PMC5028904

